# Supplementary material for: Depression among older parents’ and their associated factors after adult children’s migration: A mixed method study in an urban setting of Kathmandu Metropolitan City
Source: PLOS Ment Health. 2026 May 5;3(5):e0000239. doi: 10.1371/journal.pmen.0000239 (PMC13143126; doi:10.1371/journal.pmen.0000239)
Supplement: Attachment — Submitted filename: Rebuttal Letter.docx [file pmen.0000239.s004.docx]

**Rebuttal Letter**

**Manuscript ID:** PMEN-D-24-00540

**Journal:** PLOS Mental Health

**Title:** Depression among older parents’ and their associated factors after adult children’s migration: A mixed method study in an urban setting of Kathmandu Metropolitan City

**Submitted to:** Karli Montague-Cardoso

Executive Editor, PLOS Mental Health

**Submitted by:** Soniya Shrestha

Patan Academy of Health Sciences

[mph.soniyashrestha@pahs.edu.np](mailto:mph.soniyashrestha@pahs.edu.np)

2025/08/13

**Dear Editor,**

We are sincerely grateful to the efforts and time taken by the editorial team and reviewers in reviewing our paper, “Depression among older parents’ and their associated factors after adult children’s migration: A mixed method study in an urban setting of Kathmandu Metropolitan City”. The feedback provided have been highly useful to improve our study better and more comprehensively, and more rigorously in its methodology as a whole.

We have carefully reviewed and replied to all comments received during the peer review process. Below, we provide detailed justifications of each point, along with the changes made. We hope these changes are acceptable to the journal and make our manuscript more robust.

**Response to Editor:**

1. **Data Availability Statement**

The dataset for the quantitative findings has been included as supplementary file namely, S3_Quantitative with this manuscript for direct access. An excel file is provided containing coding information for each category of the tool.

However, the qualitative data (codebook) might compromise patient confidentiality due to sensitive information provided during interviews. After consulting with the authors, we have decided it might not be appropriate to share such sensitive information. We have however mentioned the required interview details and findings from the qualitative study within the manuscript itself.

**Response to Reviewer #1:**

We would like to express our sincere gratitude for your thoughtful and constructive feedback, which has significantly improved the quality of our manuscript.

**Comment 1:**

We thank the reviewer for this valuable observation regarding the theoretical foundation of the study and strongly agree that the manuscript would be strengthened by incorporating well-established psychological mechanisms. We acknowledge that some research has documented positive psychological adaptation to the empty nests, but the goal of this study is to specifically examine the factors that contribute to depression in this population. Focusing on the detrimental effects on mental health will help us better identify high-risk individuals and guide focused prevention and intervention efforts. In order to provide clarity, we have included a brief note in the introduction acknowledging the possibility of positive outcomes, but stressing that the current scope is purposefully restricted to the study of depression outcomes.

Also, we have updated the framework to better reflect the design and analytical methodology of the study presenting only the major variables to prevent its past impression of mediation or path analysis.

**Comment 2:**

In the original manuscript, socioeconomic status and pre-existing medical conditions were already mentioned as variables associated to depression outcomes. Nevertheless, social support networks could not be incorporated into the analysis because they were not present in our dataset. The updated manuscript now acknowledges this limitation. (Line 737-739)

**Comment 3:**

We appreciate this feedback from the reviewer. The cutoff points for mild, moderate, and severe depression based on the Geriatric Depression Scale (GDS) have now been clearly stated in the updated manuscript. The thresholds used are in line with internationally accepted GDS thresholds, ensuring comparability with earlier studies. These specifics can be found in the Results section. (Line 223-225)

**Comment 4:**

Although some independent variables are important in bivariate analysis, the original manuscript already explains why they should not be included in the final model (Lines 238–242). To put it briefly, factors with high multicollinearity (VIF > 2), including sex, occupation, and household head, were dropped. Likewise, the variable living status was dropped due to multicollinearity with marital status, despite having a strong correlation with depression (p-value: 0.001). Lastly, the final model only included variables with a VIF < 2 and a p-value ≤ 0.25 in the bivariate analysis.

**Comment 5:**

We are thankful for this feedback and have updated the results section to clearly connect the qualitative and quantitative results. The personal experiences shared in the in-depth interviews generally supported the statistical trends, for example, quantitative data identified that those who are single (widowed, divorced or separated), the odds of being likely to suffer from depressive symptoms than those who are who are married and living together with their spouses. Qualitative interviews supported these findings, highlighting depression among older, widowed parents. These connections are emphasized in the data triangulation section of the manuscript, demonstrating both convergent, divergent and also expansive findings between the two sets of data.

**Comment 6:**

We have now included the information on intercoder reliability and bias minimization in qualitative analysis in the Methodology section in the revised manuscript. (Line 137-145)

**Comment 7:**

Thank you for the comment. We have already mentioned the quantitative and qualitative sample sizes in the abstract. (Line 29-33)

**Comment 8:**

We concur that the discussion is indeed overly descriptive. It might be mainly due to the result of our study being limited to only one variable i.e., marital status being associated with the depressive symptoms in the final model. The association between marital status and depressive symptoms have been further discussed along line 690-716. We have not included other factors found highly significant with depressive symptoms in the bivariable analysis as the final model concluded only marital status to be highly significant.

Regarding mechanism of depression, we have not much focused on it as it does not fall under the scope of our study. Our study only assessed factors that might be associated to depressive symptoms.

**Response to Reviewer #2:**

We appreciate your constructive feedback, which has helped us to critically evaluate and refine our manuscript. Below are our responses to each of your several concerns:

**Abstract:**

**Comment 1:**

Thank you for your feedback. It is indeed 2.2 million absentee population. We have rectified our errors in the revised manuscript based on your evaluation.

**Comment 2:**

We have re-written the result section of the study mentioning single (widowed, divorced or separated) vs. married. Regarding the outcome variable, since we have utilized ordinal logistic regression, the results have been reported as “the odds of being likely to suffer from depressive symptoms (i.e., moderate/severe and mild vs normal)”.

**Comment 3:**

Qualitative interviews supported these findings as qualitative interviews also revealed that most empty nests parents who are single (widowed, divorced or separated) are more likely to feel depressed which can be found in the convergent findings section of the manuscript.

**Comment 4:**

Thank you for your feedback. After careful consideration, we have re-written the conclusion part of the abstract based on your recommendation.

**Introduction:**

**Comment 1:**

The definition of older population in Nepal has been mentioned in the Introduction section of the manuscript (line 57-58)

**Comment 2:**

It indeed looked confusing when mentioning 7.4% of the population in Nepal being absent and a district (Kathmandu) with 6.56% absent population be the district with highest migration. Hence, we have revised this section of the introduction for clarity. Thank you for your suggestion.

**Comment 3:**

Regarding whether absentee and migrants are the same, we have further clarified the statement in the introduction (line 60-66). The national census reports absentee population which are further divided into two categories, one, being absentee population living within the country and the other, those living abroad.

**Comment 4:**

We have made minor changes in the statement in the revised manuscript to avoid confusion stated earlier regarding financial resources and improved access to health and welfare services adversely affecting their mental health.

**Comment 5:**

We have made minor changes in the rationale section of the study. It was previously mentioned that “There is very limited evidence linking adult children’s migration to poor mental health outcomes among older parents who are left behind”. However, we have rectified the statement in the revised manuscript as there indeed is very limited evidence linking adult children’s migration to mental health outcomes among empty nests older population. Studies have been conducted among older parents with children living abroad including few comparative studies but not solely among the empty nests. The study sample is novel and fresh to the field of research. Also, this study explores the expectations of empty nests older parents following the migration of their adult children which has not been done before.

**Comment 6:**

We have added a total of 6 references as past evidences of children’s migration and left-behind parent’s mental health in the introduction section.

**Comment 7:**

The objectives previously mentioned in line 78-80 have been rectified based on the objectives section of the manuscript.

**Methodology:**

**Comment 1:** Methodology or Methods?

Thank you for your comment. We have specifically used the term ‘Methodology’ and not ‘Methods’ to better reflect the theoretical approach and chosen methods. The paper emphasizes both on how and why the specific approaches were selected not only limited to procedures and tools for data collection.

**Comment 2: Sampling frame**

Social security allowance is indeed provided to different age groups based on old age, disability, single woman or widowed. Old age allowance was provided at the age of 68 years (FY 2081/82) later changed to 70 years. Single woman allowance is provided at the age of 60 years. However, other allowances e.g. Widowed and disability are provided at any age as per need.

We were only provided with the list of beneficiaries only above the age of 60 years from the social security allowance list. This includes all the beneficiaries stated above including old age allowance- Dalits. Hence, we have not excluded anyone based on ethnicity be it Brahmins or Chhetri’s as all of the beneficiaries all included in the same list of old age allowance. However, those with incomplete contact details or those who could not be contacted during the data collection phase were excluded from the sampling frame.

**Comment 3: Number of older populations reached out**

Regarding how many older people were reached out, a total of 722 people were identified to be above 60 years receiving some form of social security allowance. Among them, 69 were deceased or could not be contacted. A total of 653 people were contacted through the provided phone numbers asked about their family status. Only those parents aged above 60 years with all their children living abroad were identified (Total identified: 203) and included in the sampling frame. Once the sampling frame was developed, Simple random sampling (SRS) was utilized and the samples were randomly selected from the list using RANDBETWEEN function in Excel (Total: 140).

**Comment 4:** **Sample size calculation**

Thank you for your comment. We have corrected the error previously made on the original manuscript. The proportion indeed is 0.018 (8.18%) however the calculated sample size stands corrected.

**Comment 5:** **Sampling and data saturation**

Judgmental sampling is another name for purposive sampling. Purposive sampling was utilized in order to ensure proper selection of participants who can offer rich, diverse perspectives which is crucial to ensure data quality.

Data saturation was ensured when no new information was received during data analysis post iterative data collection. During data analysis, similar codes were repeatedly received during coding of the transcripts and data saturation was ascertained after careful observation by 2 data analysts.

**Comment 6:** **Inclusion criteria**

The participants are indeed parents who are empty nests with all their children living abroad. Those with at least children living together are excluded from the study.

**Comment 7: Conceptual Framework**

We have updated the framework in light of this feedback to better reflect the design and analytical methodology of the study. While still clearly demonstrating the study's focus on the factors causing depression among empty-nest parents, the revised framework now presents the major variables and their proposed relationships without giving the impression of mediation or path analysis.

**Comment 8: Multivariate of Multivariable?**

Thank you for your feedback. We have corrected the analysis from multivariate to multivariable in the revised manuscript. The original study observed 2 outcomes (Depression and Loneliness) however, since this paper only assess depression outcomes, we have changed multivariate to multivariable in the revised manuscript. The same applied to bivariate.

**Comment 9: Regarding p-value of what?**

The bivariable relationship between each independent variable and the outcome variable is represented by the p-value mentioned in the statement. In the final multivariable model, only variables with a VIF < 2 and a p-value ≤ 0.25 in the bivariable analysis were kept. The updated manuscript now includes this clarification. (Line 173-175)

**Comment 10: Regarding who the supervisors are**

This manuscript indeed was prepared based on the thesis report. Regarding who are the supervisors, the authors list already mentions who are involved in the research and those who have prepared the manuscript.

**Comment 11: Abductive Coding**

To clarify on the abductive coding, it is a qualitative data analysis method that combines both inductive and deductive approaches to develop theories.

**Comment 12:** **Ethics**

Regarding how the participants were handled later does not fall under the scope of the study. Hence, it has not been included in the manuscript. However, in accordance with ethical norms, participants who scored high on the Geriatric Depression Scale (GDS) were directed to suitable mental health professionals for additional assessment and assistance. Furthermore, their answers were kept private, and their involvement had no bearing on the type of care they received.

**Comment 13: Study measures and variables section**

As the list of variables are included within the conceptual framework, we omitted the variables list section to make it look less like a thesis report as you have mentioned above.

**Comment 14: GDS measures depression or depressive symptoms?**

The Geriatric Depression Scale (GDS) measures depressive symptoms among older adults and is not a clinic diagnosis of depression.

**Results:**

**Comment 1: Numbers in parentheses mean**

The numbers in parentheses in lines 196 to 200 includes the number of cases identified for each of the chronic diseases. It was indeed unclear in the original manuscript so we have rectified in the revised one.

**Comment 2: Categories for number of children and number of children abroad**

We have mentioned <=2 categories for number of children and number of children abroad for ease of data analysis based on the mean score of the number of children. It is not mandatory to mention both however, it was grouped into only 2 categories for regression analysis.

**Comment 3: GDS and depression level categories**

The cutoff points for mild, moderate, and severe depression based on the Geriatric Depression Scale (GDS) have been clearly stated in the revised manuscript. The thresholds used are in line with internationally accepted GDS thresholds. However, through utilization of ordinal logistic regression, the results were reported as “Moderate/Severe and Mild versus Normal” which basically is automatically divided into ‘depression’ vs ‘no depression’. You can find this mentioned in the manuscript in Results section line 235-239.

**Comment 4: Binary logistic or Ordinal logistic regression?**

Ordinal logistic regression has been used for the analysis of the study as the outcome variable i.e., Depression has natural ordering (Mild, Moderate and Severe). You can clearly identify the type of logistic regression analysis utilized based on results reported for multivariable analysis in line 244-248. The results have been reported as “the odds of being more likely (i.e., moderate+ severe and mild versus normal) to suffer from depression” throughout the manuscript (also within the abstract). Since the cutoff was made as “Moderate/Severe and Mild versus Normal” which basically implies ‘depression’ vs ‘no depression’. This was included only for ease of understanding to all the viewers as results reported through ordinal logistic regression might be difficult for many of them to comprehend and interpret.

**Comment 5: Variables sex, occupation and household head being correlated**

All of the variables with VIF greater than 2 were dropped and excluded from the final model. Table 5 also shows VIF of all the variables where we can clearly see variables sex, occupation and household head have VIF of 3.36, 2.30 and 2.77 respectively which indicates multicollinearity within the variables. In order to eliminate its effects, we have dropped all the variables from the final model despite being highly corelated with p-value less than 0.05.

Also, the variable living status even though very highly correlated (p-value: 0.001) had to be dropped due to high multicollinearity with the marital status variable. The variable marital status was taken into final model.

**Comment 6: Table 5 missing?**

Table 5 is not included within the manuscript but attached as a supplementary file.

**Comment 7: p < 0.001 instead of p = 0.000**

Thank you for the feedback. We have made the changes accordingly in the revised version of the manuscript.

**Comment 8: Qualitative Results**

We concur that many of the codes were not directly related to depression. However, this paper also reports convergent, divergent and also expansive findings along with expectations of parents following migration of their children which is another objective of the study itself.

We sincerely appreciate the time and effort the reviewers and the editorial team have dedicated to evaluating our manuscript. Their valuable feedback has guided us to enhancing clarity and methodological transparency. We have worked diligently to revise the manuscript, addressing conceptual framework, study design, literature reviews, and analytical rigor. We hope these revisions sufficiently addresses the concerns raised, strengthen the study’s contribution, and reinforce its relevance. We believe the improvements demonstrate the study’s value and its suitability for publication.

We look forward to your further assessment and hope our revised manuscript meets the journal’s standards for publication.

Sincerely,

Soniya Shrestha
