# Supplementary material for: Depression among older parents’ and their associated factors after adult children’s migration: A mixed method study in an urban setting of Kathmandu Metropolitan City
Source: PLOS Ment Health. 2026 May 5;3(5):e0000239. doi: 10.1371/journal.pmen.0000239 (PMC13143126; doi:10.1371/journal.pmen.0000239)
Supplement: Attachment — Submitted filename: Rebuttal_Letter_auresp_2.docx [file pmen.0000239.s005.docx]

Rebuttal Letter

**Manuscript ID:** PMEN-D-24-00540

**Journal:** PLOS Mental Health

**Title:** Depression among older parents’ and their associated factors after adult children’s migration: A mixed method study in an urban setting of Kathmandu Metropolitan City

**Submitted to:** Karli Montague-Cardoso

Executive Editor, PLOS Mental Health

**Submitted by:** Soniya Shrestha

Patan Academy of Health Sciences

[mph.soniyashrestha@pahs.edu.np](mailto:mph.soniyashrestha@pahs.edu.np)

2026/01/09

Dear Editor,

We are sincerely grateful to the efforts and time taken by the editorial team and reviewers in reviewing our paper, “Depression among older parents’ and their associated factors after adult children’s migration: A mixed method study in an urban setting of Kathmandu Metropolitan City”. The feedback provided have been highly useful to improve our study better and more comprehensively, and more rigorously in its methodology as a whole.

We have carefully reviewed and replied to all comments received during the peer review process. Below, we provide detailed justifications of each point, along with the changes made. We hope these changes are acceptable to the journal and make our manuscript more robust.

**Response to Reviewer #1:**

We would like to express our sincere gratitude for your thoughtful and constructive feedback, which has significantly improved the quality of our manuscript.

**Comment 1:** A stronger literature review would situate parents’ mental health within the cultural realities of migration in the region. Rather than listing studies, please offer a concise synthesis that explains what we know and where findings diverge. For example, discuss how norms of filial piety, obligations around intergenerational support, and the social meaning of being a “migrant household” might increase or buffer depression risk among older parents. Consider the dual role of remittances and regular communication as potential protective resources, alongside the strain created by reduced proximal caregiving. It would help to specify what is actually limited in prior work to clarify how the present study advances the literature.

- Thank you for your comment. We revised the introduction section to provide a concise synthesis that situates parents’ mental health within the cultural context of migration, emphasizing norms of filial piety, intergenerational caregiving, and expectations of hands-on caregiving including roles of remittances and regular communication.

**Comment 2:** The introduction and discussion would also benefit from an explicit, culture-grounded theoretical framework and elaboration on the underlying mechanisms. Please explain that psychological outcomes around the “empty nest” reflect a balance between role loss and role-strain relief, and that this balance is shaped by culturally patterned factors such as familial roles and obligations and social norms about when and why children leave in collectivist culture. Discuss how remittances as moral obligations, frequency and quality of transnational contact, intergenerational co-residence, and community participation might exacerbate or buffer depression risk for parents with all children abroad. Articulating this culture-grounded framework will be useful for the readers to understand mixed findings in the literature and a clear rationale for your study. There is a lack of elaboration on the mechanisms underlying the studied relationship.

- Thank you for this important suggestion. While we agree that a culture-grounded theoretical framework would enrich interpretation, this was not explicitly incorporated at the data collection stage. The study was designed as a mixed-methods study with a sequential embedded Quan–Qual approach, primarily aimed at estimating associations rather than theorizing culturally grounded mechanisms. As such, we are unable to fully elaborate a culture-based explanatory framework (e.g., role loss versus role-strain relief) post hoc without over-interpreting the data. We have clarified this as a limitation and briefly acknowledge relevant cultural mechanisms in the discussion to contextualize our findings.

**Comment 3:** An adjusted OR of 28.3 for being single is extremely large. Consider to check for sparse cells, separation, influential cases, or mis-specification, and report model fit indices and diagnostic

- Thank you for this comment. We carefully re-examined the regression model to assess the unusually large adjusted odds ratio for being single (widowed, divorced or separated). Diagnostic checks were conducted for sparse cells, complete or quasi-complete separation, influential observations, and potential model mis-specification. These assessments did not identify any issues that would invalidate the estimate.

**Comment 4:** The results section is improved. What would strengthen the mixed-methods claim is an explicit “joint display” that positions qualitative themes alongside the corresponding quantitative findings, especially around marital status/widowhood, living arrangement, communication, and material support, so readers can see where narratives explain the patterns.

- We thank the reviewer for this constructive suggestion. To strengthen the mixed-methods claim, we have now developed a joint display that explicitly aligns the key qualitative themes with the corresponding quantitative findings. The joint display focuses on major convergent findings, in particular on marital status/widowhood, reason for migration, life satisfaction, fear of bad happening and memory problems, that might contextualize the observed statistical patterns. This has been added to the Results section to enhance the integration of the quantitative and qualitative components of the study.

**Comment 5:** The discussion should also expand more on the theoretical implications of the findings. This would help to further expand the discussion section.

- Thank you for this suggestion. We have expanded the discussion to highlight the theoretical implications of our findings. Specifically, we discuss how spousal presence provides emotional security, maintains social roles, and buffers against stress, while spousal absence—especially in empty-nest households—amplifies role loss, isolation, and psychological vulnerability.

**Comment 6:** For the qualitative component, if raw transcripts cannot be shared, consider depositing a de-identified excerpt set plus the codebook (with sensitive details masked) to meet transparency goals while protecting participants.

- Thank you for the suggestion. As raw transcripts cannot be shared due to confidentiality considerations, I will upload the codebook, with any sensitive details appropriately masked, to support transparency while ensuring the protection of participants.

**Response to Reviewer #2:**

We appreciate your constructive feedback, which has helped us to critically evaluate and refine our manuscript. Below are our responses to each of your several concerns:

**Comment 1:** Please clarify the interpretation of the ordinal logistic regression findings. As currently written (in the abstract and results section), the statement “the odds of being likely to suffer from depressive symptoms was 28.3 times …” reads like a binary logistic regression interpretation. In ordinal logistic regression, the model estimates cumulative odds. Therefore, the result should be phrased as: single individuals had 28.3 times higher cumulative odds of being in a higher category of depressive symptoms (mild or moderate/severe vs. normal, and moderate/severe vs. mild/normal) compared to married individuals, holding other variables constant.

- We agree that the previous wording reflected a binary logistic interpretation and did not accurately convey the cumulative nature of the ordinal model. We have now revised the abstract and the Results section to correctly state that the model estimates cumulative odds. Specifically, the results are now phrased as, “The study revealed that single (widowed, divorced or separated) parents had 28.3 times higher cumulative odds (p-value: 0.000, CI: 9.19-103) of being in a higher category of depressive symptoms i.e., “moderate/severe and mild” versus “normal” compared to those who are married and living together with their spouses, holding constant all other variables”.

**Comment 2:** Did you check the proportional odds (parallel slopes) assumption of ordinal logistic regression? This assumption requires that the relationship between each pair of outcome groups is the same—that is, the effect of predictors on the odds of being in a higher versus lower category is constant across thresholds. Please report whether this assumption holds.

- We thank the reviewer for raising this important point regarding the proportional odds (parallel slopes) assumption of the ordinal logistic regression model. We indeed tested this assumption using the Brant test. We conclude that the parallel assumption holds since the probability (p-values) for all variables are greater than alpha=0.05. Therefore, the proportional odds assumption is not violated and the model is a valid model for this dataset.

**Comment 3:** Please delete the phrase “As the sampling frame was unavailable for the above 60 population” (page 7, lines 111–112).

- Thank you for the comment. The phrase “As the sampling frame was unavailable for the above 60 population” has been deleted from page 7 (lines 111–112) as suggested.

**Comment 4:** My understanding is that the social security allowance is provided to older adults above 60 years of age from certain groups (e.g., ethnic minorities, Dalits, widows), but not universally to all. For example, older adults from Brahmin/Chhetri groups are eligible only from age 70. If correct, this would mean the sampling frame is not representative of all older adults. Please clarify and, if applicable, acknowledge this as a limitation.

- Yes, it indeed is correct that in Nepal the allowance is not provided universally to all individuals aged 60 and above. Rather, eligibility from age 60 applies to specific groups, including Dalits, selected marginalized ethnic groups, and widows, whereas individuals from other caste/ethnic groups (e.g., Brahmin/Chhetri) become eligible only from age 70 (previously 68). As the sampling frame was derived from the social security allowance registry, adults aged 60–69 from non-eligible groups were underrepresented. Hence, we have now explicitly acknowledged this as a limitation of the study.

**Comment 5:** Please explain the rationale for selecting the age cut-off of 68 years, as well as the cut-off of 23 years for the length of the last child’s migration (Table 2).

- The age cut-off of 68 years (during the time of data collection) was selected based on the eligibility threshold for receiving the Government of Nepal’s old-age social security allowance. The cut-off of 23 years for the duration of the last child’s migration was derived from the distribution of the variable in our dataset. The median age was found to be 23 years, hence it was selected.

**Comment 6:** If the study population consists of empty-nest older people, why is there a discrepancy between the number of children and the number of children abroad? Shouldn’t all children be out-migrated abroad? Please clarify.

- The term *empty-nest older people* in this study refers to older adults who do not have any co-residing children in the household at the time of the survey, rather than only those whose children have migrated abroad. Therefore, the children (daughters) of empty-nest older people may be living elsewhere within Nepal i.e., post marriage. As a result, the total number of children does not necessarily equal the number of children who are out-migrated abroad, which explains the observed discrepancy.

**Comment 7:** The sentence “Since the results were reported as ‘Moderate/Severe and Mild versus Normal’ which basically is automatically divided into ‘depression’ vs ‘no depression’” (page 17, lines 236–238) is unclear and should be revised for clarity.

- We have removed the sentence due to it leading to more confusion.

Example:

**Comment 8:** I recommend using the terms research team and subject experts instead of supervisor and co-supervisor.

- We thank the reviewer for the suggestion. We have revised the manuscript to replace the terms *supervisor* and *co-supervisor* with *research team* and *subject experts* throughout, to better reflect the roles and responsibilities in the study.

**Comment 9:** I also suggest having the manuscript reviewed by someone experienced in editing and proofreading academic English to improve clarity and grammar.

- Thank you for the suggestion. The research team indeed proofread the manuscript for grammar. However, we could not pursue professional editing or change the language proficiency due to economic constraints.

We sincerely appreciate the time and effort the reviewers and the editorial team have dedicated to evaluating our manuscript. Their valuable feedback has guided us to enhancing clarity and methodological transparency. We have worked diligently to revise the manuscript, addressing conceptual framework, study design, literature reviews, and analytical rigor. We hope these revisions sufficiently addresses the concerns raised, strengthen the study’s contribution, and reinforce its relevance. We believe the improvements demonstrate the study’s value and its suitability for publication.

We look forward to your further assessment and hope our revised manuscript meets the journal’s standards for publication.

Sincerely,

Soniya Shrestha
