# Supplementary material for: Depression among older parents’ and their associated factors after adult children’s migration: A mixed method study in an urban setting of Kathmandu Metropolitan City
Source: PLOS Ment Health. 2026 May 5;3(5):e0000239. doi: 10.1371/journal.pmen.0000239 (PMC13143126; doi:10.1371/journal.pmen.0000239)
Supplement: Attachment — Submitted filename: Rebuttal_Letter_auresp_3.docx [file pmen.0000239.s006.docx]

**Response to the Reviewers:**

**Reviewer #1:** We would like to sincerely thank the reviewer for their time, careful evaluation, and constructive feedback, which have greatly contributed to improving the quality of this manuscript. We have carefully revisited your feedbacks to ensure they are fully addressed and have tried our best to provide detailed clarifications and revisions in the new version of the manuscript.

1. The extremely large adjusted odds ratio for marital status (AOR ≈ 28.3) remains a substantive concern. Although the rebuttal indicates that diagnostic checks were conducted, these checks are not reported in the manuscript itself. Given the magnitude and wide confidence interval of this estimate, the manuscript should include supporting information such as a simple cross-tabulation of depression categories by marital status, clarification of any sparse cells, and at least one sensitivity analysis. That will help to improve transparency.

- Thank you for your concern. Cross-tabulation indeed indicated a small number of widowed/divorced/separated participants in the normal depression category (Table 5). To evaluate the appropriateness and stability of the ordinal logistic regression model, the proportional odds assumption was formally tested and found to hold. This supports the validity of using the ordinal model and suggests that the estimated association between marital status and depression severity is statistically reliable, although the results are interpreted with consideration of the sparse cell distribution. We have now also mentioned the finding of proportional odds assumption/parallel line test within the manuscript itself.

Since the **p-value (0.17) is greater than 0.05**, we **fail to reject the null hypothesis**.

Hence, the **proportional odds assumption holds** and the effect of marital status is consistent across outcome categories.

2. The added data triangulation section and joint display are a clear step forward and do help support the mixed-methods claim. At the moment, though, the joint display is doing most of its work around marital status. It would be even stronger if the authors could expand this table, or add a second one, to more clearly bring in living arrangements, communication patterns, and material support.

- In response to your valuable suggestion, we have expanded the joint display to incorporate findings regarding communication patterns, bringing both the quantitative results and qualitative quotes into dialogue.

Regarding the inclusion of material support (such as remittances), we carefully considered this addition. However, we were unable to include it in the joint display as it was unfortunately not a specific topic explored during the study. Consequently, we lack the corresponding qualitative data necessary to perform a meaningful methodological triangulation for this specific factor. We have added a brief note in the limitations section to acknowledge that insights on material support were not captured in this study.

We hope the expanded joint display adequately addresses your concern and enhances the overall mixed-methods presentation of the manuscript.

3. For the qualitative component, sharing the codebook is a helpful step toward transparency. That said, it would be useful if the Data Availability Statement were a bit clearer about why full transcripts cannot be shared and whether de-identified excerpts (beyond those already quoted in the paper) could be made available in some controlled way.

- Thank you for this valuable suggestion. We agree that clarity in the Data Availability Statement is important for transparency. We have now revised the Data Availability Statement to more clearly explain that full interview transcripts cannot be shared due to ethical and confidentiality considerations, as they contain sensitive personal information that could potentially lead to participant identification despite de-identification efforts. We have however added the codebook with the excerpts of the participants for data transparency.

4. The discussion has clearly been strengthened, especially the way it unpacks spousal loss and the idea of a “double loss” in empty-nest households. This interpretation comes across as thoughtful and is nicely supported by both the quantitative and qualitative results. It would be even more compelling if the authors could push this a bit further to reflect on the wider migration–care context. For example, why factors like communication or remittances did not emerge as independent predictors, despite featuring prominently in the narratives.

- Thank you for this encouraging feedback helping to deepen our analysis. We agree that contextualizing these findings within the wider transnational migration-care landscape adds critical depth to the manuscript.

In response to your suggestion, we have expanded the Discussion section to reflect on why communication did not emerge as an independent predictor of depression.

Regarding remittances, we wish to respectfully clarify that material support like remittances was not a variable measured or explored in this study. Hence, we have added a brief note in the limitations section to acknowledge that insights on material support were not captured in this study.

**Reviewer #2:**

Thank you very much for your valuable comments and constructive feedback. We sincerely appreciate the time and effort you invested in reviewing our work, which has helped us improve the quality and clarity of the manuscript. The corresponding author is a student for whom English is a second language, and want to apologize if the manuscript does not fully adhere to standard English. Nevertheless, we have made every effort to ensure that it is written as clearly and accurately as possible. Below we have tried answering to your queries as much as we could.

1. The interpretation of ordinal logistic regression still does not seem correct. In an ordered logit model with three outcome categories (as appears to be the case here), the proportional odds interpretation concerns the odds of being in a higher versus lower category across the cumulative cut-points, not a single binary comparison.
**However, the output you provided raises a more fundamental concern:**
How was the outcome ordered/coded in the ordinal model (i.e., the exact category order)? Based on the intercept labels, the ordering appears to be Mild → Moderate/Severe → No Depression, which is not the usual severity order (No Depression → Mild → Moderate/Severe). This can occur when outcome levels are left in alphabetical order rather than being explicitly set as an ordered factor. Because the meaning of a “higher category” depends entirely on the coding order, an incorrect order would make the reported coefficient/odds ratio interpretation misleading. Please re-check and clarify the coding/order used in the ordinal model.

- Thank you for your concern. It indeed occurs when the factor variable is taken into the model due to its preference to alphabetical order rather than an actual order. In order to avoid that, we specifically provided numeric codes to the labels as;

No Depression: 0,

Mild Depression: 1,

Moderate + Severe Depression: 2

We then named the variable “depression_num_new”;

Later, during analysis we specifically used the variable as a factor variable as given in the example below;

From the example given above the cutoffs shown above, the ordering now appears to be No Depression→ Mild Depression→ Moderate + Severe Depression. Hence, the odds of being in a higher versus lower category across the cumulative cut-points was made this way.

2. The output you provided corresponds to the marital status coefficient for widowed/divorced/separated (β=−2.126) and an odds ratio of 0.119 (95% CI approximately 0.057–0.240). This is not compatible with the adjusted OR reported in the manuscript (28.3; 95% CI 9.19–103). Please confirm which model/output corresponds to the results reported in Table 6 and reconcile this discrepancy.

- Apologies for the confusion created due to wrong attachment of the brant test in the previous rebuttal letter. We have further clarified the steps in your query number 4.
- However, here I have included the findings after adjusting the variables from Table 5 (Bivariate analysis).

Outcome 1:

Outcome 2:

Outcome 3:

3. I did not understand what you mean by “The age cut-off of 68 years (during the time of data collection) was selected based on the eligibility threshold for receiving the Government of Nepal’s old-age social security allowance.” Is this true? Also, “The median age was found to be 23 years, hence it was selected.” Not sure whose median age are you referring to here as 23 years?

- Regarding the first statement, the age cut-off of **68 years** was intended to reflect the eligibility criterion for the Government of Nepal’s old-age social security allowance applicable during the data collection period which has been clearly stated within the manuscript. During the data collection, the age cut-off was indeed 68 years to be eligible for old-age social security allowance which was recently increased to 70 years after the budget allocation for fiscal year 2025/26.
- However, regarding the second statement, “median age” was wrongfully written in the rebuttal letter. We apologize for the confusion. The cut-off of 23 years was clearly meant to be the **mean duration of the last child’s migration**.

4. The Brant output contradicts your claim that “p-values for all variables are > 0.05” and that the assumption holds. You can see in the stata output you provided: Omnibus test: χ2=13.29, df = 1, p = 0 and Variable-specific test (marital status): χ2=13.29, df = 1, p = 0 (p < 0.001). This means the proportional odds assumption has not met.

- We had mistakenly presented and pasted the error one previously. We apologize for the confusion. Here’s a breakdown of the model for marital status with brant test showing proportional odds (parallel slopes) assumption of the ordinal logistic regression model.

**Step 1:**

**Step 2:**

Since the **p-value (0.17) is greater than 0.05**, we **fail to reject the null hypothesis**.

Hence, the **proportional odds assumption holds** and the effect of marital status is consistent across outcome categories.

- We have also included brant test showing proportional odds (parallel slopes) assumption of the final model as response to your query number 2 which further supports the parallel slopes assumption.

5. When the software outputs p = 0.000, please report this as p < 0.001, as the probability is not literally zero.

- Thank you for the comment. Accordingly, we have revised the manuscript to report these values as **p < 0.001** throughout the text and tables to ensure correct statistical reporting.

6. “The multivariable ordinal logistic regression revealed only the variable marital status was the independent predictor of depression outcomes.” Not sure what you mean by ‘independent’ here?

- Thank you for this comment. In this context, the term “independent” was intended to indicate that marital status remained significantly associated with depression outcomes after adjusting for other covariates included in the multivariable ordinal logistic regression model.
- To avoid ambiguity, we have revised the sentence in the manuscript to state that “marital status was the only variable that remained significantly associated with depression outcomes in the multivariable model after adjustment for other variables”, replacing the term “independent predictor” for clarity.
