# Supplementary material for: Depression among older parents’ and their associated factors after adult children’s migration: A mixed method study in an urban setting of Kathmandu Metropolitan City
Source: PLOS Ment Health. 2026 May 5;3(5):e0000239. doi: 10.1371/journal.pmen.0000239 (PMC13143126; doi:10.1371/journal.pmen.0000239)
Supplement: Attachment — Submitted filename: Rebuttal_Letter_auresp_4.docx [file pmen.0000239.s007.docx]

**Manuscript ID:** PMEN-D-24-00540

**Journal:** PLOS Mental Health

**Title:** Depression among older parents’ and their associated factors after adult children’s migration: A mixed method study in an urban setting of Kathmandu Metropolitan City

**Submitted to:** Karli Montague-Cardoso

Executive Editor, PLOS Mental Health

**Submitted by:** Soniya Shrestha

Patan Academy of Health Sciences

[mph.soniyashrestha@pahs.edu.np](mailto:mph.soniyashrestha@pahs.edu.np)

2026/04/07

**Dear Editor,**

We are sincerely grateful to the efforts and time taken by the editorial team and reviewers in reviewing our paper, “Depression among older parents’ and their associated factors after adult children’s migration: A mixed method study in an urban setting of Kathmandu Metropolitan City”. The feedback provided have been highly useful to improve our study better and more comprehensively, and more rigorously in its methodology as a whole.

We have carefully reviewed and replied to all comments received during the peer review process. Below, we provide detailed justifications of each point, along with the changes made. We hope these changes are acceptable to the journal and make our manuscript more robust.

**Response to the Reviewers:**

**Reviewer #2:** Thank you once again for your continued and highly detailed engagement with our manuscript. We deeply appreciate the substantial time and energy you have dedicated to reviewing our work across four rounds of revision. Your rigorous feedback has undeniably strengthened the paper.

In this latest revision, we have carefully addressed your recent comments.

**1. Did the outputs/results of multivariable analysis in Table 6 change after adjusting the coding error of depression variable?**

- Thank you for your careful attention to our variables. We would like to clarify that there was no coding error regarding the depression variable in our underlying data or analysis. The confusion may have stemmed from how the variable's coding was initially presented in the response to your queries. Because the underlying code and data were correct from the beginning, no adjustments to the data were necessary. Consequently, the outputs and results of the multivariable analysis presented in Table 6 are accurate as originally calculated and have not changed. We have used numeric coding for the ‘depression’ variable (used the variable as a factor variable) as discussed in the previous revision. The slightest changes in the manuscript have been made with track changes to highlight the changes we have made.
- However, one single change has been made for a variable, ‘family type’ where the reference variable was inverted. It has further been discussed in point number 3 below.

**2. The title and footnotes of Table 6 should be revised to explicitly state that the estimates are derived from an ordinal (proportional odds) logistic regression model, as the current presentation may still be misinterpreted as binary logistic regression.**

- Thank you for this suggestion. While we had explicitly detailed our use of an ordinal (proportional odds) logistic regression model in the Methodology, we appreciate you highlighting the need for greater clarity here. We have revised both the title and the footnotes of Table 6 to explicitly state that the estimates are derived from an ordinal (proportional odds) logistic regression model.

**3. The adjusted odds ratio reported for family type (nuclear) appears inconsistent with the ordinal logistic regression output, as it does not reflect the exponentiated model coefficient and reverses the direction of effect; this estimate should be recalculated and corrected.**

- Thank you for your careful attention to this estimate. Upon reviewing the output and the table, we realized that the confusion stemmed from how we assigned the reference category in the manuscript versus how the software parameterized it. In our initial Table 6, we had set 'Nuclear' as the reference category and mathematically inverted the software's output to report the odds for 'Joint' families (1/0.498 = 2.01). However, you are entirely correct that reporting it this way makes it appear inconsistent with the raw exponentiated model coefficient, which used 'Joint' as the baseline and calculated the odds for 'Nuclear' (0.498). To eliminate any confusion and ensure our table directly reflects the raw model output, we have updated Table 6. We have now set 'Joint' as the reference category, and correctly reported the adjusted odds ratio for 'Nuclear' family type as 0.498 (95% CI: 0.18–1.39). Thank you for helping us improve the clarity of our reporting.

**4. How did you calculate the bivariate (unadjusted) odds ratios in Table 6? They also need to be based on separate ordinal logistic regression models, this should be stated explicitly (e.g., using polr(depression ~ predictor, …..)), as unadjusted estimates from cross‑tabulations or binary models would not be directly comparable to the multivariable ordinal results.**

- Thank you for raising this important methodological point. We completely agree that the unadjusted estimates must be derived from the same model type to be directly comparable to the multivariable ordinal results.
- We have indeed calculated the bivariate (unadjusted) odds ratios using ordinal (proportional odds) logistic regression models for each predictor, exactly as you have recommended. You can find the details of bivariate (unadjusted) odds ratios in Table 5 which has been attached as a supplementary file titled, ‘S2 Table 5. Bivariable analysis of the study’.
- To assure you of our analytical approach, we have attached a screenshot of the raw statistical output for two of the variables ‘marital status’ and ‘ethnicity’ for your reference. Here, you can observe the unadjusted odd ratio to be 44.1 (16.7-142) for ‘marital status’ which has been mentioned in table 6 accordingly.

Example 1: ‘marital status’

Example 2: ‘ethnicity’

- Bivariate analysis for other variables was done in similar way.

**5. Please correct any instances where p = 0.000 has been rewritten as p = 0.001; the appropriate convention is p < 0.001.**

- Thank you for this correction. We have updated all instances where p-values were incorrectly reported as p = 0.001 (or previously shown as p = 0.000 in software output) to the correct convention of p < 0.001.

**6. It looks like you used R for analysis, but you mentioned STATA?**

- We utilized a combination of both software packages for different phases of the study. All descriptive statistics and initial data management were conducted using both Stata and EZR as STATA helps in proper visualization of frequency and percentages. These were compared to findings in EZR for verification. However, the inferential statistics—including the ordinal logistic regression models—were performed using EZR.

**7. Because you mentioned that there were wrong outputs attached at multiple instances, I highly recommend to upload the raw data and analysis code in the supplementary file for transparency and reproducibility purpose. This would further ensure the integrity of this important research.**

- Thank you for your comment. We are happy to confirm that the fully anonymized raw dataset has actually already been uploaded and attached as a Supplementary File (S3 Data. Quantitative data of the study) in our previous submission, and we have ensured it remains attached for this final version.

Given the extensive refinement this manuscript has undergone through your ongoing guidance, we believe it has now reached its strongest possible form. We feel confident that all underlying concerns have been thoroughly addressed.

Thank you again for your exhaustive efforts in helping us bring this paper to the finish line.

Sincerely,

Soniya Shrestha
